# Evaluation of the Total Mercury Weight Exposure Distribution Using Tree Bark Analysis in an Artisanal and Small-Scale Gold Mining Area, North Gorontalo Regency, Gorontalo Province, Indonesia

**DOI:** 10.3390/ijerph19010033

**Published:** 2021-12-21

**Authors:** Hendra Prasetia, Masayuki Sakakibara, Koichiro Sera, Jamie Stuart Laird

**Affiliations:** 1 Graduate School of Science and Engineering, Ehime University, Matsuyama 790-8577, Japan; 2 Department of Forestry, Faculty of Agriculture, University of Lampung, Bandar Lampung 35145, Indonesia; 3 Research Institute for Humanity and Nature, Kyoto 603-8047, Japan; sakaki@chikyu.ac.jp; 4 Faculty of Collaborative Regional Innovation, Ehime University, Matsuyama 790-8577, Japan; 5 Cyclotron Research Center, Iwate Medical University, Tomegamori 348-58 Tomegamori, Takizawa 020-0173, Japan; ksera@iwate-med.ac.jp; 6 School of Chemistry, University of Melbourne, Parkville, VIC 3010, Australia; jslaird@unimelb.edu.au

**Keywords:** atmospheric, mercury, ASGM, amalgamation, accumulation, tree bark

## Abstract

It is well known that atmospheric mercury (Hg) contaminates air, water, soil, and living organisms, including trees. Therefore, tree bark can be used for the environmental assessment of atmospheric contamination because it absorbs heavy metals. This study aimed to establish a new biomonitoring for the assessment of atmospheric Hg pollution. Reporting on atmospheric Hg contamination in an artisanal and small-scale gold mining (ASGM) area in North Gorontalo, Indonesia, we calculated the total weight of Hg (THg) and quantitatively measured the concentrations of Hg in the tree bark of *Mangifera indica*, *Syzygium aromaticum, Terminalia catappa,* and *Lansium domesticum*. The THg of Hg in the *M. indica* tree bark samples ranged from not detected (ND) to 74.6 μg dry weight (DW) per sample. The total Hg in the tree bark of *S. aromaticum, T. catappa,* and *L. domesticum* ranged from ND to 156.8, ND to 180, and ND to 63.4 μg DW, respectively. We concluded that topography significantly influences the accumulation of Hg together with local weather conditions. A mapped distribution of the THg suggested that the distribution of THg in the tree bark was not affected by the distance to the amalgamation site. Therefore, tree bark can be used as biomonitoring of atmospheric Hg contamination for the assessment of ASGM areas.

## 1. Introduction

Artisanal and small-scale gold mining (ASGM), which provides income to many poor communities in developing countries, such as Indonesia, uses several gold extraction methods that use mercury (Hg). The International Labor Organization estimates that there are currently around 13 million artisanal miners in 55 countries [1]. During the processes of panning and amalgamation when amalgam is burned in a small charcoal fire, ASGM releases Hg into the atmosphere [2,3]. ASGM is a widely recognized major source of Hg contamination, and its activities cause serious Hg pollution.

Mercury is extremely dangerous and contaminates air, water, soil, and living organisms. The health of miners and people living within or outside ASGM areas is affected by the inhalation of atmospheric Hg [3]. Anthropogenic Hg emissions to the atmosphere significantly interfere with the natural Hg cycle [4]; however, estimates of natural global Hg emissions vary by orders of magnitude [4,5]. The increasing total weight of Hg (THg) in the soil is likely due to the deposition of Hg released into the atmosphere [6].

Plants are sensitive to their environmental conditions, and their elemental compositions actively reflect changes in these conditions [7,8,9]. Tree bark, in particular, can be used to assess the status of the environment, especially the level of Hg contamination, and sources of pollution can be traced by the enrichment of trace elements in tree bark [10]. Airborne particles, trapped within the structure of tree bark, accumulate over several years [11]. The uptake of trace elements by plants involves both root uptake and foliar absorption, including from the deposition of particulate matter on leaves [12]. Different plant uptake patterns are based on three factors: plant species, element species, and conditions at specific sites [13]. Canopy crops act to trap gaseous and particulate Hg, which can then be trapped by tree bark depending on its roughness and porosity [14].

Although the use of tree bark has been studied for environmental pollution assessments, corresponding atmospheric contamination has not been comprehensively discussed in relation to the distance from the source of the contamination and the transport of Hg in the atmosphere. Consequently, the practical application of tree bark as a biomonitoring for the atmosphere has not been previously proposed. Therefore, several tropical species, including *Mangifera indica, Syzygium aromaticum, Terminalia catappa,* and *Lansium domesticum*, were comprehensively studied to establish a new biomonitoring for the assessment of atmospheric Hg pollution in an ASGM area in North Gorontalo Regency, Gorontalo Province, Indonesia.

## 2. Materials and Methods

### 2.1. Sampling Plots

**Figure 1:**
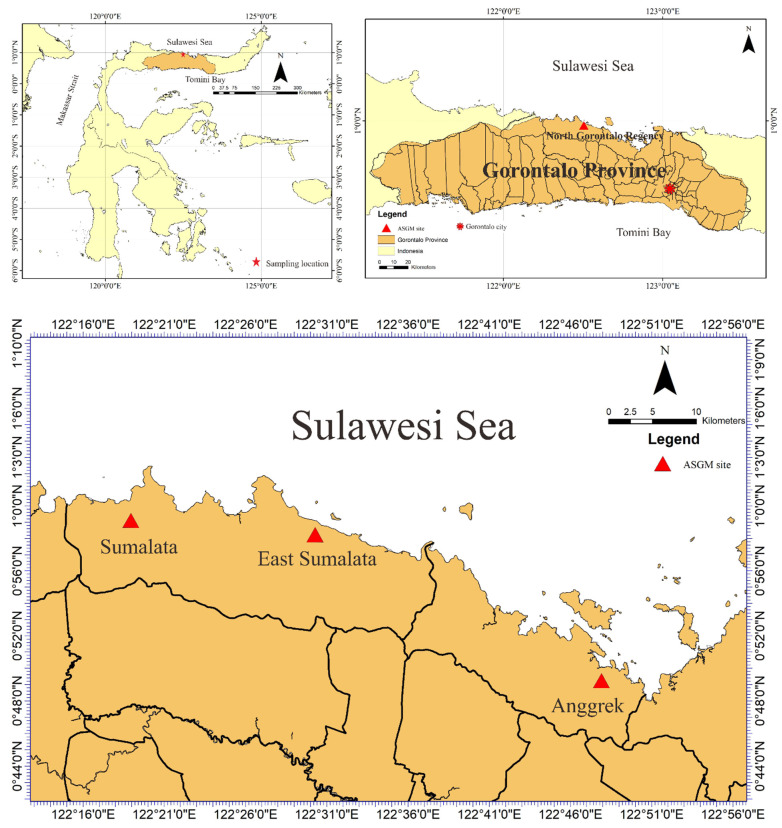
Artisanal and small-scale gold mining (ASGM) sites in North Gorontalo Regency, Gorontalo Province, Indonesia.

**Figure 2:**
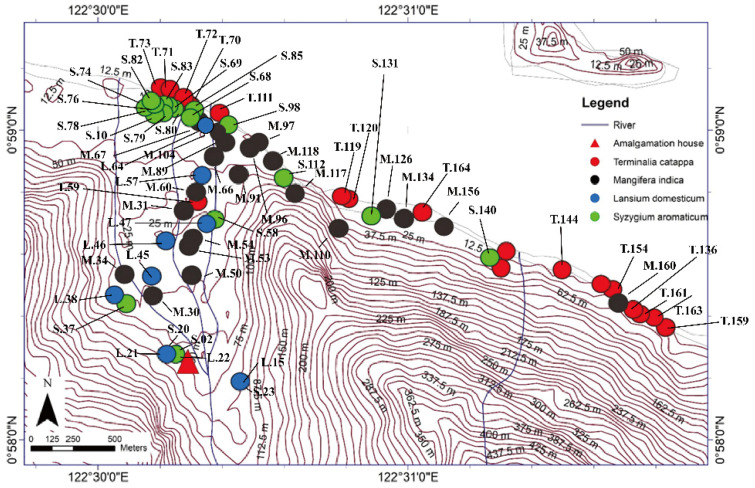
*Terminalia catappa, Mangifera indica, Lansium domesticum,* and *Syzygium aromaticum* sampling points in East Sumalata District, Gorontalo Province, Indonesia (N = 65). The sampling point coordinates was measured using a GPS (Oregon 650 TCJ; Garmin), and the map contours were created by ArcGIS 10.3 and Global Mapper 10 software.

We performed a field survey and laboratory analyses to determine heavy metal concentrations (particularly Hg) in tree bark to assess the environmental contamination in the study area. This study research was conducted during August and September 2016, entering the rainy season in Indonesia, in an ASGM area of north Gorontalo Regency. In this regency, there are three ASGM sites, which are located in different districts, shown in Figure 1. The study area of this research was located in east Sumalata District, shown in Figure 1. This study was obtained tree bark about 65 samples in total with details 21 samples of *M. indica*, 20 samples of *S. aromaticum,* 15 samples of *T. catappa,* and 9 samples of *L. domesticum* from the study area, as shown in Figure 2, and sampled randomly selected that were around settlements area. The mercury is very harmful to humans and, therefore, the tree barks samples, mostly found in the inhabitant’s yard, were selected in this study due to its capability as biomonitoring of atmospheric Hg contamination. These sampling species were grown naturally in this area, which dominated in the lower topography of this area.

The tree bark samples were collected from 1.3 m above the ground, being the diameter at breast height standard height. The bark was collected as 10 × 10 cm fragments to ensure homogeneous sampling. About 12–18 cm^2^ of 100 cm^2^ bark samples were analyzed to indicate the Hg concentrations.

### 2.2. Analytical Methods

The tree bark samples were dried at ~80 ℃ for 2 days in a ventilated oven. About 12–18 cm^2^ of each sample was crushed to a fine powder with a powder mill (Varian PM-2005 m, Osaka Chemical Co., Ltd., Osaka, Japan) to produce homogeneous samples for particle-induced X-ray emission (PIXE) analysis. The tree bark powders (30 mg) were then digested by a mixture of indium (In) and HNO_3_ in a ratio of 3:100 before the heavy metal concentrations, such as Pb, Zn, Fe, Hg, and As, were determined by PIXE [15,16,17] at Iwate Medical University (Iwate, Japan). The dimensions of the tree bark samples were calculated by ImageJ, Version 1.48 software. The analytical conditions followed [15]. A small cyclotron provided a 2.9 MeV-proton beam on the target after passing through a beam collimator of graphite. The maximum beam intensity on the target was approximately 40 nA for a beam spot diameter of 2 mm and 80 nA for a diameter of 6 mm. Elements from Na to U were detected by two ORTEC Si (Li) detectors. The elements heavier than Ca were detected by the first detector, which had a 0.025 mm-thick Be window and a 6 mm active diameter, with X-rays with an energy resolution of 154 eV at 5.9 keV and a 300 to 500 µm thick Mylar absorber inserted between the target and the detector. The other low atomic number elements were detected by the second detector, which had a 0.008 mm Be window and a 4 mm active diameter, a resolution of 157 eV, and a small graphite aperture without an absorber.

### 2.3. Calculation of THg

The bioaccumulation of Hg was estimated using the THg, defined as the dry weight of the sample multiplied by the Hg concentration determined by the PIXE analysis in 100 cm^2^ of sample [17].

THg = (DW × C_Hg_) × (FD/real square) (1)

where DW is the dry weight of the sample, C_Hg_ is the Hg concentration, FD is fragment dimensions (100 cm^2^), and real square is the sample dimension, as measured by ImageJ.

### 2.4. Statistical Analysis

Statistical analyses were performed using IBM SPSS Statistic 21 for Microsoft Windows. The Shapiro–Wilk test was used to check the normality of the Hg concentrations. The data were log-normally distributed, so the Kruskal–Wallis ANOVA test was used to test for significant differences, with *p* < 0.05 considered statistically significant.

## 3. Results

### 3.1. Estimation of THg

**Figure 3:**
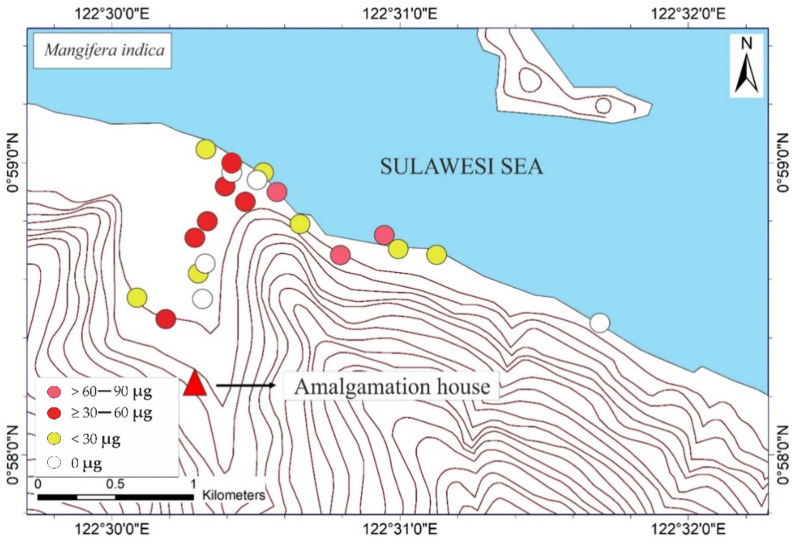

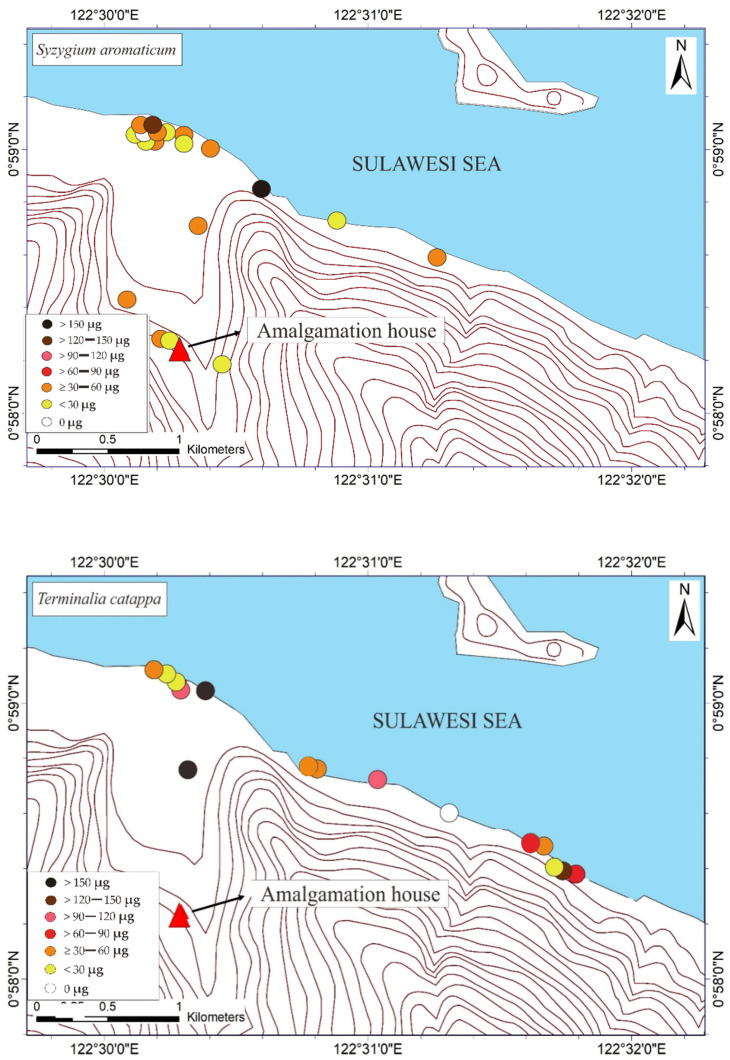

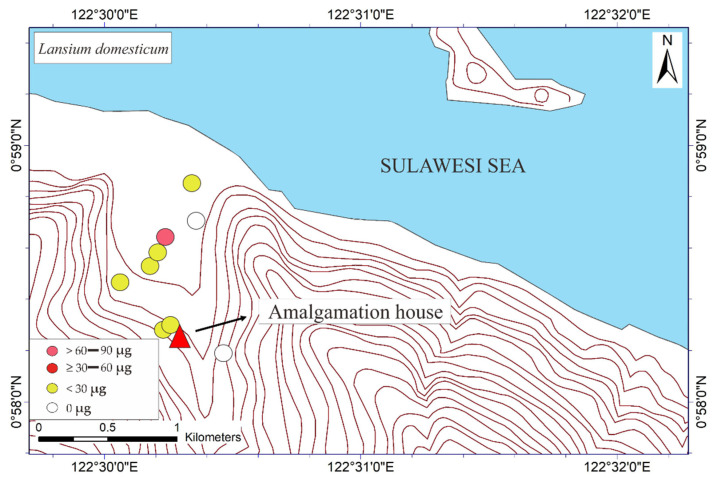
Mapped distribution of total weight of Hg (THg) in the bark of *Mangifera indica*, *Syzygium aromaticum*, *Terminalia catappa*, and *Lansium domesticum* (*N* = 65).

The THg in the *M. indica* bark samples ranged from not detected (ND) to 74.6 μg DW (see Table 1). The ND results were probably attributable to the absorption of Hg due to different weather conditions under different topographic conditions. The THg in the bark samples of *S. aromaticum, T. catappa,* and *L. domesticum* ranged from ND to 156.8, ND to 180, and ND to 63.4 μg DW, respectively (see Table 2, Table 3 and Table 4). The average results of THg showed that the *T. catappa* has highest mean of THg about 66.2 μg DW. Then, it was followed by *S. aromaticum, M. indica,* and *L. domesticum* with THg concentrations about 42.4, 26.1, and 15.4 μg DW, respectively. The highest THg was located in areas of lower topography, as shown in Figure 3. This suggested that atmospheric Hg contamination is most dominant in estuaries. A plant is categorized as toxic if the concentration of Hg exceeds 1 ppm [9], so based on this study results, the bark of *M. indica, S. aromaticum, T. catappa* and *L. domesticum* could be used as a biomonitoring of atmospheric Hg contamination in the environmental assessments of ASGM areas.

## 4. Discussion

### 4.1. Mapping Distribution of THg in ASGM Area

**Figure 4:**
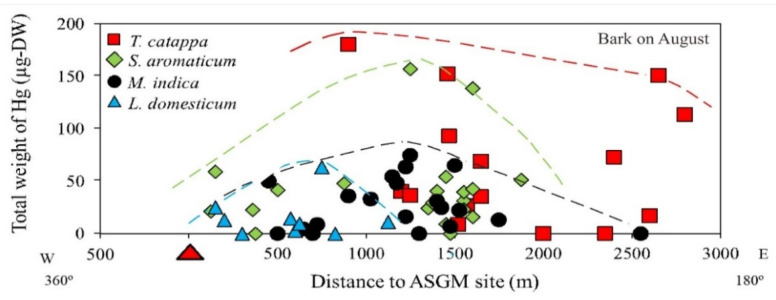
Total weight of Hg (THg) in the bark of various tree species.

The mapped distribution of THg in the bark of *M. indica, S. aromaticum, T. catappa* and *L. domesticum* (Figure 3) suggested that topography significantly influences the accumulation of Hg in the atmosphere together with local weather conditions. However, the distribution was not affected by the distance to the amalgamation site, as shown in Figure 3 and Figure 4. This is probably attributable to the wind direction, which transports and deposits the atmospheric Hg in the estuary area. The concentrations on the tree barks were affected by atmospheric attachment to the barks, not from the root absorption in soil and/or water [17].

Zang et al.’s [18] study showed that Hg transport and dispersion in the atmosphere may not always be explained by so-called prevailing winds. In their study area, there was no prevailing wind strong enough to control the direction of the Hg diffusion in the atmosphere, so the evenly distributed Hg in their bark samples was attributed to precipitation rather than dry deposition or contamination from a reservoir. Local influences can diminish the relative importance of the general atmospheric transport of Hg [18]. According to Barnes et al. [19], tree bark accumulates metals as a function of its proximity to the pollution source.

Elemental Hg is the predominant (95%) form in the atmosphere, with reactive and particulate forms also being important [20]. Plants have been shown to accumulate atmospheric elemental Hg (Hg^o^) in foliage over time as a function of exposure to concentrations of Hg in the air and soil [21,22]. Elemental Hg is thought to be directly taken up via stomata and/or cuticles and possibly transformed into a water-soluble Hg compound and absorbed via leaves [23,24]. In addition to the concentration of atmospheric Hg, environmental factors, such as solar irradiation, air temperature, altitude, and biological factors, such as plant species, leaf age, and leaf placement, also significantly influence the uptake of Hg by foliage [22,25,26,27].

In addition, climate change has the potential to alter the sequestration of Hg from forest soils via direct pressures (meteorological) or indirect pressures (vegetation changes) [28]. This could have indirect consequences for forests that may also affect Hg cycling. According to Richardson and Friedland [28], vegetation type can affect many aspects of Hg cycling in forest soils. The variable foliar morphology and biomass characteristics of different vegetation types can affect Hg levels in litterfall. The physical attributes of the canopy structure of each species can also directly affect the accumulation of Hg in foliage [29,30].

### 4.2. Distribution of THg Based on Distance to Source and Elevation

The distance of the samples from the ASGM site did not influence the THg attachment; however, the total weight of Hg in the bark taken from *T. catappa* was higher compared to *S. aromaticum*, *M. indica*, and *L. domesticum,* as shown in Figure 4. In the study area, *T. catappa* and *M. indica* grow naturally, whereas *S. aromaticum* and *L. domesticum* are cultivated for economic purposes.

Living at lower topographic levels along the coastline, *T. catappa* is mainly an estuary plant, and some of the *S. aromaticum* sampled in this study was also cultivated at lower topographic levels along the coastline. As shown in Section 3.1, the highest THg was found in the *S. aromaticum* and *T. catappa,* located in the coastline area.

A wide range of total Hg concentrations in the air has been reported in the literature. The latitudinal distribution of total gaseous Hg indicates a background level of about 2 ng m^−3^ in the lower troposphere of the northern hemisphere and just over 1 ng m^˗3^ in the southern hemisphere, at least in an oceanic environment [31]. In general, elemental Hg seems to be the dominant form [31]. The Hg associated with aerosol particles normally makes up only a small fraction of the total airborne Hg; however, the role of particulate Hg in the atmospheric is important. The atmospheric cycle retains Hg in the atmosphere for long periods, and, consequently, transports it over very long distances [32]. Mercury vapor, which comprises 95–99% of total Hg in the atmosphere, has an atmospheric residence time of 1 year [4], allowing for global dispersal and the contamination of ecosystems through both wet and dry deposition [33,34].

### 4.3. Distribution of THg Based on Tree Species

**Figure 5:**
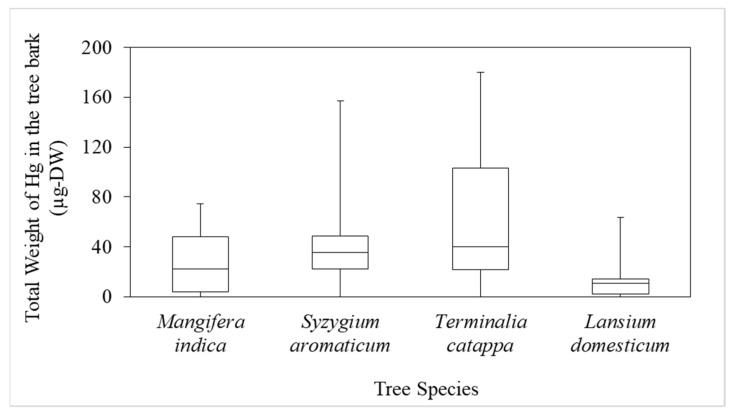
Boxplot of the total weight of Hg (THg) in the bark of the various species.

A THg boxplot for the various tree species showed that the mean values for *T. catappa, S. aromaticum, M. indica,* and *L. domesticum* were 66.2, 42.4, 26.1, and 15.4 µg-DW, respectively, but there were no significant differences (*p* < 0.05), as shown in Figure 5. This indicated that tree species significantly influenced the attachment of Hg to the bark. We assumed that *T. catappa bark* has a high porosity, so it retains Hg better than the other species.

## 5. Conclusions

Our results showed that there was a high level of heterogeneity in the THg in the bark of both the naturally grown and cultivated tropical tree species we studied. The sampled *M. indica* and *T. catappa* were located in areas of lower topography on the coastline of the study area. *M. indica* is a native plant mostly cultivated close to houses as a garden plant. *S. aromaticum* and *L. domesticum* are native plants cultivated for economic purposes in the study area. This variability suggested that topography significantly influenced the accumulation of Hg together with local weather conditions, but was not affected by distance from the amalgamation site. This study indicated that tree species significantly influenced the attachment of Hg to the bark. We assumed that *T. catappa bark* has a high porosity, so it retains Hg better than the other species. We found that the tree bark of *M. indica, S. aromaticum, T. catappa* and *L. domesticum* could be used as a biomonitoring of atmospheric contamination assessment in the ASGM area.

## Figures and Tables

**Table 1:** Total weight of Hg (THg) in the bark and diameter of *Mangifera indica*.

No	Sample	T(Hg) (µg—DW) ± SD	Diameter (cm)
1	*M. indica* 30	49.8 ± 27.7	42.7
2	*M. indica* 117	15.9 ± 15.3	40.1
3	*M. indica* 118	64.5 ± 20.1	46.8
4	*M. indica* 156	13.0 ± 14.3	25.5
5	*M. indica* 126	63.5 ± 49.1	30.3
6	*M. indica* 160	ND	19.1
7	*M. indica* 134	22.2 ± 31.4	27.4
8	*M. indica* 53	8.70 ± 16.4	20.7
9	*M. indica* 66	47.9 ± 31.1	69.7
10	*M. indica* 96	ND	23.9
11	*M. indica* 31	35.5 ± 34.6	72.6
12	*M. indica* 89	ND	36.3
13	*M. indica* 97	24.3 ± 12.3	89.2
14	*M. indica* 104	54.1 ± 27.3	47.8
15	*M. indica* 34	4.10 ± 7.10	41.4
16	*M. indica* 67	6.60 ± 13.7	11.8
17	*M. indica* 54	ND	29.9
18	*M. indica* 91	30.9 ± 15.0	24.2
19	*M. indica* 110	74.6 ± 27.6	43.9
20	*M. indica* 60	32.9 ± 23.4	58.3
21	*M. indica* 50	ND	41.1
	Mean	26.1 ± 17.4	40.1

T: Total; DW: Dry Weight; SD: Standard Deviation; ND: Not Detected.

**Table 2:** Total weight of Hg (THg) in the bark and diameter of *Syzygium aromaticum*.

No	Sample	T(Hg) (µg—DW) ± SD	Diameter (cm)
1	*S. aromaticum* 23	20.9 ± 28.9	11.1
2	*S. aromaticum* 79	9.10 ± 18.2	39.8
3	*S. aromaticum* 140	51.0 ± 33.7	19.1
4	*S. aromaticum* 68	24.2 ± 28.5	16.2
5	*S. aromaticum* 76	16.0 ± 20.6	33.4
6	*S. aromaticum* 69	31.2 ± 48.5	10.8
7	*S. aromaticum* 82	138 ± 68.7	18.5
8	*S. aromaticum* 112	156.8 ± 79.6	18.5
9	*S. aromaticum* 85	23.0 ± 17.7	13.7
10	*S. aromaticum* 20	58.8 ± 33.3	23.9
11	*S. aromaticum* 98	54.3 ± 26.5	25.5
12	*S. aromaticum* 10	ND	28.4
13	*S. aromaticum* 02	22.6 ± 10.2	29.8
14	*S. aromaticum* 83	39.5 ± 27.4	26.1
15	*S. aromaticum* 58	47.8 ± 19.6	10.5
16	*S. aromaticum* 78	ND	16.9
17	*S. aromaticum* 74	42.6 ± 16.3	17.2
18	*S. aromaticum* 37	41.3 ± 23.5	11.1
19	*S. aromaticum* 80	40.6 ± 24.4	30.6
20	*S. aromaticum* 131	29.6 ± 15.3	11.5
	Mean	42.4 ± 27.0	20.3

T: Total; DW: Dry Weight; SD: Standard Deviation; ND: Not Detected.

**Table 3:** Total weight of Hg (THg) in the bark and diameter of *Terminalia catappa*.

No	Sample	T(Hg) (µg—DW) ± SD	Diameter (cm)
1	*T. catappa* 71	8.70 ± 22.0	17.5
2	*T. catappa* 120	35.9 ± 17.9	20.7
3	*T. catappa* 136	68.4 ± 26.3	55.1
4	*T. catappa* 144	ND	17.8
5	*T. catappa* 163	150 ± 42.4	43.6
6	*T. catappa* 73	35.4 ± 38.7	32.8
7	*T. catappa* 164	113 ± 69.7	50.3
8	*T. catappa* 59	180 ± 105	44.9
9	*T. catappa* 72	26.4 ± 43.5	41.7
10	*T. catappa* 70	92.7 ± 43.6	36.6
11	*T. catappa* 159	72.5 ± 56.4	58.9
12	*T. catappa* 119	40.1 ± 69.4	53.8
13	*T. catappa* 154	ND	89.5
14	*T. catappa* 161	16.8 ± 19.2	53.8
15	*T. catappa* 111	152 ± 63.3	31.5
	Mean	66.2 ± 41.2	43.2

T: Total; DW: Dry Weight; SD: Standard Deviation; ND: Not Detected.

**Table 4:** Total weight of Hg (THg) in the bark and diameter of *Lansium domesticum*.

No	Sample	T(Hg) (µg—DW) ± SD	Diameter (cm)
1	*L. domesticum* 26	25.0 ± 18.8	38.5
2	*L. domesticum* 57	ND	34.1
3	*L. domesticum* 64	10.7 ± 14.7	37.6
4	*L. domesticum* 38	2.50 ± 15.5	37.9
5	*L. domesticum* 45	14.1 ± 25.1	38.5
6	*L. domesticum* 15	ND	30.7
7	*L. domesticum* 47	63.4 ± 26.8	27.4
8	*L. domesticum* 21	13.0 ± 13.6	10.4
9	*L. domesticum* 46	9.50 ± 25.0	10.5
	Mean	15.4 ± 15.5	29.5

T: Total; DW: Dry Weight; SD: Standard Deviation; ND: Not Detected.
